# Case of Extensive Fracture of the Skull, with Laceration of the Membranes, and Escape of Considerable Portion of the Brain, Successfully Treated

**Published:** 1827-09

**Authors:** J. C. Cox

**Affiliations:** Member of the Royal College of Surgeons, London, and of the Royal Medical Society of Edinburgh.


### INJURIES OF THE HEAD.

*Case of extensive Fracture of the Skull, with Laceration of the Membranes, and Escape of considerable Portion of the Brain, successfully treated.* By Mr. J. C. Cox, Member of the Royal College of Surgeons, London, and of the Royal Medical Society of Edinburgh.

Friday, January 6th.—George Selman, aged twenty, a healthy young man, of a sallow complexion, was working in a coal-pit, when a heavy stone fell from a height of fourteen or fifteen yards, and struck him down to the ground. He was taken up, bleeding profusely from the head, and was carried home. My assistant, who was near at the time of the accident, saw him very shortly, and sent for me.

Although he was completely stunned by the blow, and at first in a state of complete stupor, yet he soon recovered some degree of consciousness. When I saw him, he was not insensible; his countenance was natural, his pulse firm and steady. He had been bled by my assistant before my arrival.

I found a large portion of the upper and anterior part of the left parietal bone driven in by the blow. The fracture extended across the coronal suture, and a part of the frontal bone was included in the depressed portion. The bones were so fractured by the blow, that, after sawing through a small part with Hey's saw, I was able to elevate the depression, and remove the fractured portions, which measured nearly three inches long by two inches wide, but which were broken into ten pieces. The bleeding from the surface of the brain was very considerable. On raising the depressed bone, there was a gush of venous blood, and an artery of the dura mater threw out its blood vigorously. It was necessary to secure this vessel by a ligature, which was of fine silk, and cut off close to the knot. A considerable quantity of brain, estimated at a table-spoonful, had escaped, and some was still adhering to the hair. The young man retained his consciousness during the operation: he occasionally expressed a feeling of pain, and watched his brother walking in the garden, he being placed opposite the window. It was yet necessary to examine whether there were any spiculæ of bone remaining in the wound, and the finger passed into a frightful cavity in the substance of the brain. The angles of the wound were now approximated to each other by straps of sticking plaster, and the centre was dressed with pledgets of lint. After he was removed from the table into bed, he lay in a very tranquil state; his pupils dilated and contracted readily; there was no moaning or expression of suffering.

Evening.—Pulse ninety, strong; had spoken since I left him. He lay quite tranquil; pupils natural; breathing slow and regular; had made water; expressed great aversion to being disturbed.

Poultice applied to the head. V.S. ad ℥xxx. Ordered an aperient.

7th, morning.—Has passed a tranquil night; lies very still; he speaks little, but is evidently conscious of what passes around him. The wound looks well; pulse 108.

Ordered a Calomel powder, and a mixture with Salts and Senna every four hours.

Evening.—The medicines have acted on the bowels. Pulse ninety, strong; in other respects the same as in the morning.

V.S. ad ℥ xvj. Repeat the mixture.

8th.—Passed a tranquil night, but at about seven o'clock this morning became restless; face flushed; pulse 108, not strong; tongue white; skin hot; he occasionally moans. The wound looked well. He knew me when I addressed him.

℞. Calomel gr. ij.; Pulv. Jalap. gr. viij. fiat pulv. quartâ quâque horâ sumendus, cum Misturæ Cathartic cyatho ad alvum bené solvendam.

The action of the aperient removed all the unpleasant symptoms. The patient continued going on tranquilly. There was a healthy suppuration established; but, on the fifth day after the accident, a hernia cerebri began to protrude.

The poultices were omitted, and the wound dressed with dry lint, and pressure employed by straps of adhesive plaster.

The protrusion continued for some days to increase, in spite of the means employed, till it was nearly as large as the egg of a turkey, and burst open a considerable portion of the wound which had united.

The pressure was increased by means of a bandage, which was firmly bound over the projecting part.

The patient continued without any other bad symptoms of importance. He occasionally became restless and a little delirious, but an active aperient always relieved him.

At the end of three weeks, the protruded portion was evidently diminished; there was an extremely fetid discharge. The pressure employed by the bandage, &c. produced a decided effect on the tumor: if that were intermitted or diminished from any cause, the protrusion was increased; but perseverance in the system at length completely repressed it. The granulations quickly filled up the wound, and at the end of two months it was perfectly healed; and it is surprising how little mark remains of the severe injury that was sustained. His intellectual powers are not at all impaired; and he grew very fat during the confinement. He is now able to work at his usual employment.

*Observations.*—The injury sustained in this case was so considerable, that few could have anticipated any other than a fatal result; and the favourable progress of the case is to be attributed partly to the health and vigour of the patient, and also in part to the employment of active depletion in the early stage.

The occurrence of hernia cerebri after fractures of the skull is a great aggravation of the danger, not only as proving considerable injury to the membranes of the brain, but also as being frequently followed by deep-seated abscess.

There can be no doubt, however, that the evils of this accident are best averted by gentle treatment; by taking care to avoid inflammation, by strict antiphlogistic diet and venesection; and at the same time endeavouring to restrain the tumor by firm pressure. The treatment formerly adopted, of cutting away the protruded portion, employing escharotics, &c. only tends to excite that irritation which it is so important to avert, and does not cure the disease: the protrusion still occurs, and the surgeon is baffled and disappointed. At the same time deep-seated inflammation and abscess is a very common consequence of such measures. One other observation suggests itself, which is confirmed by the experience of all practical surgeons,—viz. that the evils arising from such an accident can only be averted by full and active depletion at the very early stage of the case; and the benefits of keeping up a free and regular action on the bowels can be sufficiently appreciated only by those to whom such cases are familiar.

Does not the fact of the intellectual faculties being so little impaired by such extensive injury and loss of cerebral substance, afford some confirmation to the theory of Gall and Spurzheim, that the organs by which the intellectual faculties are manifested are double?

11, *Clapton-terrace*; 1827.

